# Subsequent thyroid disorders associated with treatment strategy in head and neck cancer patients: a nationwide cohort study

**DOI:** 10.1186/s12885-019-5697-y

**Published:** 2019-05-16

**Authors:** Chien-Liang Lin, Shang-Yin Wu, Wen-Tsung Huang, Yin-Hsun Feng, Ching-Yi Yiu, Wei-Fan Chiang, Sheng-Yow Ho, Sheng-Hsiang Lin

**Affiliations:** 1 Division of Hematology and Oncology, Chi-Mei Medical Center, Liouying Campus, Tainan, Taiwan; 2 Min-Hwei Junior College of Health Care Management, Tainan, Taiwan; 3 Division of Hematology and Oncology, National Cheng Kung University Hospital, Tainan, Taiwan; 4 Division of Hematology and Oncology, Chi-Mei Medical Center, Tainan, Taiwan; 5 Division of Otolarygology, Head and Neck Surgery, Chi-Mei Medical Center, Liouying Campus, Tainan, Taiwan; 6 Division of Oral Maxillo-facial Surgery, Chi-Mei Medical Center, Liouying Campus, Tainan, Taiwan; 7 Division of Radiation Oncology, Chi-Mei Medical Center, Liouying Campus, Tainan, Taiwan; 8 Institute of Clinical Medicine, College of Medicine, National Cheng Kung University, 138, Shengli Road, Tainan, Taiwan; 9 Department of Public Health, College of Medicine, National Cheng-Kung University, Tainan, Taiwan; 10 Biostatistics Consulting Center, National Cheng Kung University Hospital, College of Medicine, National Cheng-Kung University, Tainan, Taiwan

**Keywords:** Head and neck cancer, Treatment strategy, Thyroid disorders, Cohort study

## Abstract

**Background:**

We investigated the risk of thyroid disorders, namely hypothyroidism, thyrotoxicosis and thyroiditis, in head and neck cancer patients undergoing multimodal treatment.

**Methods:**

A cohort study design using Taiwan’s National Health Insurance Research Database was used to assess head and neck cancer patients over 20 years old. The cohort was divided into one group who underwent primary tumor excision only (PTE) and another with additional neck dissection (PTE + ND). The tumor sites were stratified to estimate the tumor-site-specific risk of thyroid disorders. The effect of subsequent resurgery, radiotherapy (RT), chemotherapy (CT), and concomitant (CCRT) or sequential chemoradiation therapy (sequential CT+ RT) on the risk of thyroid disorders was explored.

**Results:**

For 1999–2012, 7460 patients who underwent PTE + ND and 3730 who underwent PTE were enrolled and followed-up until the end of 2013. There were 122 and 50 patients in the two groups, respectively, who developed thyroid disorders, with no statistical difference between the groups. Patients with hypopharyngeal, oropharyngeal, or laryngeal cancer in the PTE + ND group had a higher risk of thyroid disorders (adjusted HR: 1.50, 95% CI: 0.67–3.38) than those in the PTE group when adjusted for covariates and mortality. Patients who underwent subsequent RT (adjusted HR: 3.64, 95% CI: 1.05–2.77) and CCRT (adjusted HR: 1.70, 95% CI: 1.05–2.77) after PTE + ND had a significantly higher risk of thyroid disorders.

**Conclusion:**

RT results in a major risk of subsequent thyroid disorders, and ND may exacerbate this effect. Physicians should monitor thyroid function from two years after treatment initiation, especially in patients who undergo ND and subsequent RT.

**Electronic supplementary material:**

The online version of this article (10.1186/s12885-019-5697-y) contains supplementary material, which is available to authorized users.

## Background

In 2015, head and neck cancer accounted for a substantial proportion of the entire cancer burden globally, with 410,304 new cases of oral cavity cancer and 161,467 of pharyngeal cancer [1]. In that year in the United States, there were 49,670 new cases of and 9700 deaths from oral cavity and pharyngeal cancer [2]. In Taiwan, where betel-nut chewing and cigarette smoking are endemic, the age-standardized incidence of and mortality from head and neck cancer are 20.88 and 7.53 per 100,000 persons, respectively, and the disease is ranked sixth in incidence and fifth in mortality among all cancers [3]. Planning comprehensive treatment strategies for head and neck cancer patients requires multidisciplinary teams incorporating surgeons, radioncologists, and medical oncologists, and the cost of such treatment comprises a substantial proportion of the country’s annual medical cost burden. After a patient has undergone a series of treatments, long-term complications such as xerostomia, osteoradionecrosis, trismus, dysphagia, ototoxicity, peripheral neuropathy, chronic nephropathy, and thyroid disorders can negatively affect their quality of life. Thyroid disorders in particular require careful surveillance, but can be corrected by medical treatment. If not sufficiently monitored, such disorders can progress to chronic diseases such as atherosclerosis, coronary artery diseases, and dyslipidemia, and can also affect wound healing [4–6].

The probability of hypothyroidism (the most thoroughly investigated thyroid disorder) after radiotherapy (RT) is in the range 7.5–38%, depending on the population and RT dosage [7–14]. Patients with nasopharyngeal cancer seem to have a higher risk of hypothyroidism than patients with any other type of head or neck squamous cell carcinoma, because of differences in the RT field and dosage [15]. With respect to surgical procedures, the effects of laryngectomy, thyroidectomy, and neck dissection (ND) on the probability of hypothyroidism has been discussed in the literature. Laryngeal and hypopharyngeal cancer, as well as laryngectomy, which occur in close proximity to the thyroid gland, are thought to lead to hypothyroidism after RT [8, 16–18]. However, Sinard et al. also reported hypothyroidism in 11% of head and neck cancer patients who had undergone nonlaryngeal surgery [14]. All types of ND apart from partial and radical thyroidectomy are expected to cause long-term sclerosis of the neck, because of alterations in the distribution of the blood supply, leading to possible subsequent thyroid problems; however, two studies have found no significant effects on hypothyroidism in subgroup analyses [14, 19].

Recently, taxane and cetuximab have been administered in conjunction with traditional chemotherapy (CT), with promising efficacy in squamous cell carcinomas of the head and neck in a neoadjuvant or definitive setting. Although there is as yet little evidence regarding the long-term effects of these new agents, a study by Diaz et al. found that 61/128 (47.7%) locally advanced head and neck cancer patients who had received intensity-modulated RT concurrent with taxane-based CT developed hypothyroidism [20].

The diversity of results regarding hypothyroidism is exacerbated by the difficulty of diagnosing it consistently: a wide variety of methods are used to detect thyroid function, and there is a large range of healthy thyroid-stimulating hormone (TSH) levels. Due to the fact that hypothyroidism is ill-defined based on laboratory data alone, some small-scale studies have investigated this issue by using questionnaires administered to patients presenting overt symptoms after they had undergone multimodal treatments [17, 18].

The associations between ND, subsequent thyroid disorders, and different modalities of treatment used in conjunction with ND, are uncertain. For this reason, we compared the risk of three thyroid disorders—hypothyroidism, thyroiditis, and thyrotoxicosis—between head and neck cancer patients who underwent ND and those who did not, and also investigated the effects of subsequent cancer treatments.

## Methods

### Database

We retrieved the medical information for patients identified as having a catastrophic illness from Taiwan’s National Health Insurance Research Database (NHIRD) between 1 January 1997 and 31 December 2013. The NHIRD was constructed by the National Health Research Institutes (NHRI) to contribute to the Registry for Catastrophic Illness Patient Database. When patients are recognized as having a catastrophic illness, based on pathological reports and image studies conducted by specialists, they are entitled to reimbursements of medical expenses from the National Health Insurance (NHI) of Taiwan. Basic information for each patient, such as age, sex, insured date and area, occupation, and income, is recorded in the database, along with at least two diagnoses from an outpatient department (OPD) and three upon admission, using ICD-9-CM codes for every patient visit. Details of drug administration, interventions, and examinations are also recorded in this database. To conform to the rules of the Personal Information Protection Act, scrambled numbers were assigned to each patient as a surrogate for their true identity. Our study was therefore granted an exemption from full review by the Institutional Review Board (IRB) of the Chi-Mei Medical Center (Application number: 10701-E02).

### Study design and subjects

**Fig. 1:**
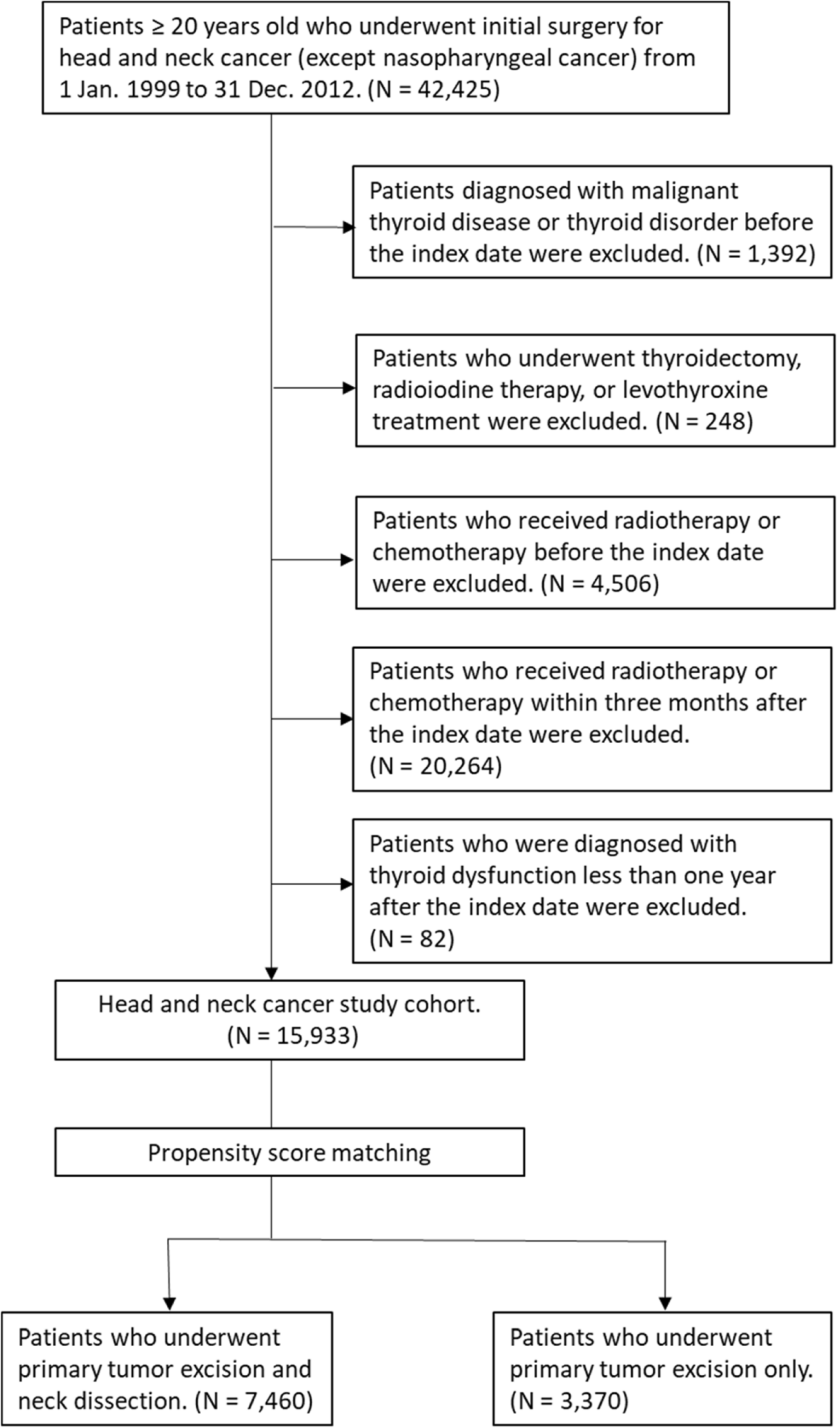
Study flow chart

This retrospective cohort study aimed to investigate the association between the treatment strategy for head and neck cancer and thyroid disorders. The inclusion criteria for the study were that the patient: 1) had to be over 20 years of age, 2) had to have been diagnosed with head and neck cancer (except nasopharyngeal cancer, ICD-9-CM code 147) and registered in the catastrophic illness database, and 3) had to have undergone surgery as the initial cancer treatment between 1999 and 2012. The exclusion criteria were as follows: 1) diagnoses of thyroid neoplasm disease or thyroid disorders before the index date; 2) history of thyroidectomy, or radioiodine or levothyroxine treatment; or 3) history of RT or CT for any reason before the index date. The index date was defined as the day of the primary surgery to treat the head and neck cancer. In order to explore the effect of different surgical procedures on subsequent thyroid function it was necessary to confine the cohort to those who had not received adjuvant RT or concomitant chemoradiation therapy (CCRT), so patients who received RT or CT less than three months after the index date were also excluded. Subjects with subsequent RT or CCRT more than three months after the primary surgery caused by their relapse disease were enrolled in the study. However, patients whose thyroid disorders occurred within one year were excluded, to avoid ill-defined cause–effect relationships (Fig. 1).

The cohort was divided into two groups: patients who underwent primary tumor excision combined with neck dissection (PTE + ND), and those who underwent primary tumor excision alone (PTE). The two groups were stratified according to type of cancer: oral cancer (ICD-9-CM: 140, 141, 143, 144, 149.8, 149.9); salivary gland cancer (ICD-9-CM: 142); oropharyngeal, hypopharyngeal, and pharyngeal cancer (ICD-9-CM: 146, 148, 149.0, 149.1); and cancer of unspecified parts of the mouth (ICD-9-CM: 145). The risk of thyroid disorders was then compared between the two groups. We also investigated the effect of subsequent strategies for treating disease recurrence after primary surgery on the risk of developing thyroid disorders. Secondary surgery for disease relapse was defined as surgery performed for head and neck cancer or neck lymphadenectomy at least three months after the index date. Subsequent RT or CT was defined as administration of these therapies at least three months after the primary surgery.

### Matching

Because of the differences in the baseline characteristics of the PTE + ND and PTE groups, propensity score matching was used to ensure comparability between both groups. For each patient, the propensity score was estimated using a logistic regression in which the covariates of sex, age, living area, enrollee category, monthly income, urbanization, and Charlson comorbidity index (CCI) were modeled. Although age and monthly income are continuous variables, they were divided into categories in this study, as described below.

### Covariates

All covariates, including basic individual information such as sex, age, living area, enrollee category (EC), monthly income, urbanization of the city of residence, and comorbidities, were obtained from the database. Patients’ ages were stratified into three groups: 20–44, 45–59, and ≥ 60 years. Four ECs were defined, based on occupation and source of income, as follows: EC1, civil servants; EC2, employees of private enterprises or institutions; EC3, self-employed individuals, other employees, and members of farmers’ or fishermen’s associations; and EC4, low-income families and unemployed pensioners. Socioeconomic status was determined based on EC, monthly income, and urbanization of the city of residence. Income was also stratified: ≤ NT$ 15,840, NT$ 15,841–25,000, and ≥ NT$ 25,001. Generally, the most urbanized cities, with the highest population densities, are located in northern Taiwan, followed by central and then southern Taiwan. We combined eastern Taiwan and the offshore islands due to their small populations. The comorbidities of each patient, which were registered as the main or secondary diagnosis at every visit to an OPD or admission within one year of the index date, were retrieved from the database to estimate the CCI. The comorbidities of interest were chronic heart, cerebrovascular, lung, liver, gastrointestinal, and kidney diseases, diabetes with or without complications, malignant disease with or without distant metastasis, and HIV infection [21]. We stratified our cohort into three CCI groups: 0–2, 3–4, and ≥ 5.

### Main outcome measurements

The primary endpoint of the present study was subsequent thyrotoxicosis (ICD-9-CM: 242.x), hypothyroidism (ICD-9-CM: 243, 244.x), thyroiditis (ICD-9-CM: 245.x), or other thyroid function disorders (ICD-9-CM: 246.3, 246.8, 246.9). The above thyroid disorder diagnoses were defined as the main outcome following a single admission or two visits to an OPD. All patients were followed until occurrence of the main outcome, death, or the end of 2013.

### Validation

We validated the ICD-9-CM codes used to identify the diagnoses of thyroid disorders in the present study using the Admission and OPD Claims databases at the Chi-Mei Medical Center, Liouying Campus, which is an 870-bed regional teaching hospital in southern Taiwan. Records in 2010 were retrieved from these databases for 200 patients randomly selected according to a ratio approximately matching that observed in the general populace. We confirmed the diagnosis of hypothyroidism as patients who underwent screening for elevated serum TSH. The definitive diagnosis of thyrotoxicosis included depressed TSH, elevated thyroxine (T4) or triiodothyronine (T3), anti-thyroid peroxidase antibody (anti-TPO Ab) screening, and sonography for thyroid morphology. For thyroiditis, the diagnosis was determined either from the pathological report or from the patient’s clinical data, and was based on elevated erythrocyte sedimentation rate (ESR), positive anti-TPO Ab or antithyroglobulin antibodies (anti-Tg Ab), and a sonographic report of the thyroid gland, with or without a record of thyroid pain.

The 200 randomly selected patients included 83 with thyrotoxicosis, 91 with hypothyroidism, and 26 with thyroiditis. Of these, 165 had a confirmed definitive diagnosis corresponding to the ICD-9-CM code defined by the National Health Insurance Administration Ministry of Health and Welfare. The positive predictive value was 82.5% (95% confidence interval: 76.4–87.4).

### Statistical analyses

After propensity score matching, baseline characteristics of the two groups were compared using the Chi-square test and Fisher’s exact test. Differences in the risk of subsequent thyroid disorders between the two groups were estimated using the Cox proportional hazard regression model, with adjustments for covariates. The appropriate test for each covariate was conducted to test the proportional hazards assumption. All variance-inflation factors for the covariates were low, indicating that no multicollinearity existed among these variables. A competing risks model was used, whereby death served as a competing risk to thyroid disorders. To determine the risk of thyroid disorder associated with different types of head and neck cancer, the patients were stratified into four groups based on tumor site: oral cancer; salivary gland cancer; combined oropharyngeal, hypopharyngeal, and pharyngeal cancer; and cancer in an unspecified site. Finally, the effect of subsequent treatment strategies (resurgery, RT, and CT) on the risk of thyroid disorders was also investigated. All statistical analyses were performed using SAS 9.3 software (SAS Institute, Cary, NC, USA).

## Results

Our 1999–2012 cohort included a total of 15,933 subjects, which was reduced to 11,190 after propensity score matching: 7460 patients in the PTE + ND group and 3730 in the PTE group (Table 1). The largest age-group of patients was 45–59 years, followed by ≥60 years and 20–44 years, which contained similar numbers. The largest proportion of the patients lived in southern Taiwan and in the least-urbanized cities. With respect to socioeconomic status, most of the patients were classified as EC3 and their monthly income fell within the intermediate range. The percentage of the patients in the PTE + ND group with CCIs in the range 0–2, 3–4, or ≥ 5 was 46.35, 31.73, and 21.92%, respectively, and this distribution was similar to that of the PTE group. The proportion of patients who received subsequent therapy was higher in the PTE + ND group than in the PTE group.

**Table 1:** Demographic characteristics of head and neck cancer patients who underwent surgery with or without neck dissection between 1999 and 2012

	PTE + ND (N = 7460)	PTE (N = 3730)	
	N (%)	N (%)	*P*
Male	6500 (87.13)	3113 (83.46)	< 0.001
Age (years)			
20–44	1465 (19.64)	783 (20.99)	0.08
45–59	3237 (43.39)	1543 (41.37)	
≥ 60	2758 (36.97)	1404 (37.64)	
Region of Taiwan			0.80
North	2504 (33.57)	1271 (34.08)	
Central	1666 (22.33)	836 (22.41)	
South	2759 (36.98)	1347 (36.11)	
East and offshore	531 (7.12)	276 (7.40)	
Enrollee category			0.24
1	565 (7.57)	314 (8.42)	
2	2410 (32.31)	1227 (32.90)	
3	3765 (50.47)	1818 (48.74)	
4	720 (9.65)	371 (9.95)	
Monthly income			0.004
≤ NT$ 15,840	2127 (28.51)	1139 (30.54)	
NT$ 15,841–25,000	3997 (53.58)	1873 (50.21)	
≥ NT$ 25,001	1336 (17.91)	718 (19.25)	
Urbanization Level			0.02
1 (most urbanized)	1868 (25.04)	999 (26.78)	
2	1890 (25.34)	860 (23.06)	
3 (least urbanized)	3702 (49.62)	1871 (50.16)	
Charlson comorbidity index			0.91
0–2	3458 (46.35)	1739 (46.62)	
3–4	2367 (31.73)	1168 (31.31)	
≥ 5	1635 (21.92)	823 (22.06)	
Subsequent therapy			
Surgery	2176 (29.17)	704 (18.87)	< 0.001
Radiotherapy	1890 (25.34)	529 (14.18)	< 0.001
Chemotherapy	1544 (20.70)	387 (10.38)	< 0.001

*PTE* primary tumor excision, *ND* neck dissection

Up until the end of 2013, 122 and 50 patients were diagnosed with subsequent thyroid disorders in the PTE + ND and PTE groups, respectively (Table 2, Additional file 1: Figure S1). The competing risk hazard was 1.25 (95% CI: 0.90–1.74). The median period between the index date and occurrence of the thyroid disorder in these 172 subjects was 3.56 years (interquartile range [IQR]: 2.06–5.48 years). When stratified according to cancer sites, patients with oropharyngeal, hypopharyngeal, or laryngeal cancer had a significantly higher risk of developing thyroid disorders than patients with other types of head and neck cancer, according to the competing risks regression (CRR) model (2.81; 95% CI: 1.37–5.75), but not according to the adjusted HR analysis (1.50; 95% CI: 0.67–3.38).

**Table 2:** Risk of thyroid disorders among head and neck cancer patients who received various surgical procedures stratified by primary tumor site

	All neck cancers		Oral cancer		Salivary gland cancer		Oropharyngeal, hypopharyngeal, and pharyngeal cancer		Cancers in unspecified sites	
	PTE + ND (N = 7460)	PTE (N = 3730)	PTE + ND (*N* = 3530)	PTE (*N* = 1237)	PTE + ND (*N* = 85)	PTE (*N* = 453)	PTE + ND (*N* = 413)	PTE (*N* = 596)	PTE + ND (*N* = 3422)	PTE (*N* = 1444)
	N (%)	N (%)	N (%)	N (%)	N (%)	N (%)	N (%)	N (%)	N (%)	N (%)
Thyroid disorders	122 (1.64)	50 (1.34)	65 (1.84)	20 (1.62)	1 (1.18)	8 (1.77)	22 (5.33)	10 (1.68)	34 (0.99)	12 (0.83)
Crude HR	1.16 (0.83–1.61)	1	1.15 (0.70–1.90)	1	0.74 (0.09–5.94)	1	1.59 (0.75–3.36)	1	1.19 (0.62–2.30)	1
Adjusted HR	1.21 (0.87–1.68)	1	1.08 (0.65–1.78)	1	0.69 (0.09–5.52)	1	1.50 (0.67–3.38)	1	1.22 (0.63–2.35)	1
CRR	1.25 (0.90–1.74)	1	1.10 (0.67–1.82)	1	0.66 (0.08–5.23)	1	2.81 (1.37–5.75)^*^	1	1.17 (0.61–2.27)	1

*PTE* primary tumor excision, *ND* neck dissection, *HR* hazard ratio, *CRR* competing risks regression

*P < 0.05

With respect to subsequent treatment, 2176 patients in the PTE + ND group and 704 in the PTE group received resurgery, of whom 41 and 11, respectively, eventually developed thyroid disorders (Table 3). Neither the PTE + ND (CRR: 1.03; 95% CI: 0.71–1.51) nor the PTE group (CRR: 1.23; 95% CI: 0.63–2.41) exhibited an increased risk of thyroid disorders, however, indicating that resurgery had no impact on the thyroid.

**Table 3:** The effect of subsequent surgery, following primary tumor excision and/or neck dissection, on thyroid disorders in head and neck cancer patients

	PTE + ND		PTE	
	No re-surgery (*n* = 5284)	Re-surgery (*n* = 2176)	No Re-surgery (*n* = 3026)	Re-surgery (*n* = 704)
	N (%)	N (%)	N (%)	N (%)
Thyroid disorders	81 (1.53)	41 (1.88)	39 (1.29)	11 (1.56)
Crude HR	1	1.11 (0.76–1.62)	1	1.05 (0.54–2.04)
Adjusted HR	1	1.06 (0.72–1.55)	1	1.11 (0.57–2.17)
CRR	1	1.03 (0.71–1.51)	1	1.23 (0.63–2.41)

*PTE* primary tumor excision, *ND* neck dissection, *HR* hazard ratio, *CRR* competing risks regression

Each group was then stratified into five categories: patients who did not receive subsequent treatment (non-RT, non-CT), RT only, CT only, CCRT, and sequential CT + RT, to examine the effect of these therapies on the risk of thyroid disorders. In the PTE + ND group, 5319 (71.3%) patients received no further treatment, 597 (8%) received RT only, 251 (3.36%) received CT only, 1133 (15.19%) received CCRT, and 160 (2.14%) received sequential CT + RT (Table 4). Compared to the group that received no further treatment, the RT only (adjusted HR: 3.64; 95% CI: 2.28–5.81) and CCRT groups (adjusted HR: 1.70; 95% CI: 1.05–2.77) had a higher risk of thyroid disorders. The median period between the index date and the occurrence of the thyroid disorders was 3.48 years (IQR: 2.45–4.65) in the RT only group and 3.49 years (IQR: 2.16–5.12) in the CCRT group. In the PTE group, however, the risk of thyroid disorders was not higher in the groups receiving subsequent treatment than in the group that did not.

**Table 4:** The effect of subsequent radiotherapy and/or chemotherapy on the risk of developing thyroid disorders in head and neck cancer patients

	PTE + ND					PTE				
	Non-RT & Non-CT (*N* = 5319)	RT only (*N* = 597)	CT only (*N* = 251)	CCRT (*N* = 1133)	Sequential CT + RT (*N* = 160)	Non-RT, Non-CT (*N* = 3079)	RT only (*N* = 264)	CT only (*N* = 122)	CCRT (*N* = 239)	Sequential CT + RT (*N* = 26)
	N (%)	N (%)	N (%)	N (%)	N (%)	N (%)	N (%)	N (%)	N (%)	N (%)
Thyroid disorders	71 (1.33)	25 (4.19)	2 (1.64)	22 (1.94)	2 (1.25)	41 (1.33)	5 (1.89)	0 (0.00)	4 (1.67)	0 (0.00)
Crude HR	1	3.70 (2.34–5.83)^*^	0.58 (0.14–2.35)	1.75 (1.08–2.82)^*^	1.10 (0.27–4.47)	1	1.47 (0.58–3.71)	–	1.47 (0.53–4.12)	–
Adjusted HR	1	3.64 (2.28–5.81)^*^	0.58 (0.14–2.36)	1.70 (1.05–2.77)^*^	1.01 (0.25–4.14)	1	1.41 (0.56–3.58)	–	1.51 (0.54–4.23)	–
CRR	1	3.00 (1.89–4.77)^*^	0.49 (0.12–2.02)	1.19 (0.74–1.93)	0.73 (0.18–2.98)	1	1.21 (0.48–3.05)	–	1.09 (0.38–3.11)	–

*PTE* primary tumor excision, *ND* neck dissection, *RT* radiotherapy, *Non-RT* non-radiotherapy, *CT* chemotherapy, *Non-CT* non-chemotherapy, *CCRT* concomitant chemoradiation therapy, *HR* hazard ratio, *CRR* competing risk regression

*P < 0.05

## Discussion

The present study is, to our knowledge, the first national, large-scale cohort study to investigate the relationship between multimodal treatment for head and neck cancer and subsequent thyroid disorders in Asia. Because thyroid disorders are chronic complications that occur after treatment, a long follow-up period was necessary, which is a strength of our study. In addition, the occurrence of thyroid disorders other than hypothyroidism after multimodal treatment has not been widely discussed.

Tell et al. analyzed 391 non-thyroid head and neck cancer patients who received various doses of RT and reported that 52 of them (13.3%) had overt hypothyroidism, which was the most comprehensively studied thyroid disorder [22]. In their multivariate analysis, a high TSH level before treatment, surgery involving the thyroid gland, and bilateral neck irradiation were risk factors for hypothyroidism. Notably, the proportion of hypothyroidism in oral cavity cancer patients was 19.4%, which is not less than that in patients with larynx or oropharynx cancer, and 17.7% of patients who underwent non-thyroid surgery developed hypothyroidism. In another prospective study, in which 251 patients were divided into six groups (non-laryngectomy, partial laryngectomy, and total laryngectomy, each with or without RT), an overall incidence of hypothyroidism of 15% was reported [14]. In that study, hemithyroidectomy and RT administered to the remaining lobe were the major factors leading to hypothyroidism, while ND and additional CT were not. Patients who underwent total laryngectomy with RT were at a significantly higher risk of hypothyroidism than those without RT. Merely using ICD-9-CM codes as surrogates for the diagnosis of thyroid disorders, as we did in the present study, may underestimate the prevalence relative to the laboratory data used in previous studies. Notably, however, a previous study that also used the NHIRD database determined the incidence of hypothyroidism to be 4.41 per 1000 person-years in non-nasopharyngeal head and neck cancer patients after RT, which is substantially lower than reported in other studies (7.5–47.7%) [15]. In the present study, which excluded subjects who underwent surgery involving the thyroid gland, patients with oropharyngeal, hypopharyngeal, or laryngeal cancer had a tendency to develop thyroid disorders after ND. Although the crude and adjusted HR suggested that the difference was nonsignificant, the CRR revealed a markedly higher risk, as a result of taking the competing risk of death into account. Subsequent RT or CCRT for disease relapse also increased the risk of thyroid disorders.

In our study, RT was the main factor triggering hypothyroidism, which is consistent with the published literature. The follicular epithelium cells of the thyroid gland are damaged by the radiation, which is thought to reduce the quantities of hormones released [23]. Radiation can recruit lymphocytes to the thyroid bed, as well as stimulate the formation of autoantibodies, leading to thyrotoxicosis or thyroiditis in vitro [24]. One study reported 30 patients with Grave’s hyperthyroidism and six with thyrotoxicosis out of 1677 Hodgkin’s lymphoma patients treated with RT at Stanford University Medical Center, with a mean 9.8-year follow-up period [25]. Other studies have also reported other thyroid disorders that are induced by RT, such as Hashimoto’s thyroiditis and other nonspecific thyroiditis with various incidence rates [26, 27]. This inspired our investigation of other thyroid disorders. In addition, radiation-induced atherosclerosis of the carotid artery, resulting in thyroid disorders, has been reported [28]. As observed in our study, ND itself may exacerbate the effect of radiation on thyroid function, presumably as a result of the fibrous changes in the skin and the sclerosis of arteries following surgery.

With respect to the timing of the occurrence of thyroid disorders after initial treatment, the reported range varies from six months to 2.03 years [12, 14, 19, 20, 22, 29–31]. The majority of these previous studies used the patients’ laboratory serum data to define their thyroid disorders according to a wide range of TSH levels, which could represent both overt and subclinical hypothyroidism. In the present study, the use of ICD-9-CM codes as the indication of diagnosis resulted in a longer period before the occurrence of the thyroid disorders. Based on the process of validation between the NHIRD database and our hospital, the majority of patients with a diagnosis of hypothyroidism or thyrotoxicosis received medication (88.75 and 100%, respectively), which indicates that only overt cases were registered in the NHIRD database. Although the function of the thyroid gland changes dynamically after receiving direct RT, not all patients with elevated TSH present with overt symptoms requiring medication [30]. Our study was thus based on a more exact measure of the incidence and timing of overt thyroid disorders following treatment.

Until now, there has been no evidence that CT induces thyroid disorders. Mercado et al. retrospectively accessed the data from a randomized trial and showed that administration of cisplatin and fluorouracil concurrently with RT did not increase the risk of hypothyroidism relative to RT alone [29]. Sinard et al. analyzed 66 of 198 head and neck cancer patients who underwent surgery with or without initial RT and then received concurrent or subsequent CT, and demonstrated that these treatment strategies had no effect on thyroid function [14]. In our study, patients in the PTE + ND group who underwent subsequent RT had a higher risk of thyroid disorders than those who underwent subsequent CCRT with similar observation time, due to their greater mean age, which meant they were unsuitable for CCRT (Additional file 2: Tables S1 and S2). In contrast, the anti-EGFR agent, cetuximab, which has been approved for concomitant use with RT by the NHI of Taiwan since July 2009, has not to our knowledge yet been proven to affect the thyroid gland. However, the number of patients in our cohort who were given cetuximab for CCRT was small, so additional cases are required to investigate this issue further.

Some authorities recommend that thyroid function should be checked as early as one month after treatment has been completed, then at three- to six-month intervals for more than five years [32]. We agree with this follow-up schedule, based on the mean occurrence of thyroid disorders 3.56 years after the primary surgery in the present study. In our opinion, in addition to TSH levels, T3 and T4 levels should be monitored regularly. Anti-TPO and anti-Tg antibodies, ESR, and sonography of the thyroid gland were checked occasionally in the present study, based on the patient’s manifestations.

There were some limitations in the present study. First, the AJCC stage of cancer was not available in the NHIRD database, which may lead to confounding by indication. However, the subjects we selected were mainly confined within early stage [33, 34]. Second, though the information of radiation field and dosage was not available in this database, the radiation dose would be ranged from 60 to 72 Gy because of the subjects in present study received adjuvant or definitive setting. Third, since no laboratory data for the patients are recorded in the database, diagnosing thyroid disorders based merely on the ICD-9-CM code may lead to an underestimation of their occurrence.

## Conclusion

In present study, patients who underwent PTE and ND followed by RT or CCRT in response to disease relapse had the greatest likelihood of developing subsequent thyroid disorders requiring treatment. Physicians should be reminded to monitor thyroid function in patients with non-nasopharyngeal cancer from two years after the initiation of treatment, especially in those who received subsequent RT. Various conditions can occur, such as hypothyroidism, thyrotoxicosis, and thyroiditis, and it is important to detect these early in order to treat them effectively.

## Additional files

Additional file 1: **Figure S1.** The cumulative incidence of subsequent thyroid disorders in PTE and PTE+ ND groups were present as solid and dotted curve, respectively. In our study design, the subjects with thyroid disorder occurred within one year after allocation were excluded, which is to prevent ill-defined cause-effect relationship, leading to no outcome obtained in the first year. (DOCX 219 kb)

Additional file 2: **Tables S1 and S2.** The Table S1 showed the similar observation time in RT and CCRT groups of subjects received PTE + ND initially. In Table S2, subjects with higher age level distributed in RT group than in CCRT group. Both tables explained the RT group had higher risk of subsequent thyroid disorder than CCRT in subjects who received PTE + ND initially didn’t cause by longer follow-up time but by older age level distribution. (DOCX 16 kb)

## Abbreviations

AJCC: American Joint Committee on Cancer
CCI: Charlson comorbidity index
CCRT: Concomitant chemoradiation therapy
CI: Confidence interval
CRR: Competing risks regression
CT: Chemotherapy
EC: Enrollee category
EGFR: Epidermal growth factor receptor
IQR: Interquartile range
IRB: Institutional Review Board
ND: Neck dissection
NHI: National Health Insurance
NHIRD: National Health Insurance Research Database
NHRI: National Health Research Institutes
OPD: Outpatient department
PTE: Primary tumor excision
RT: Radiotherapy
Sequential CT+ RT: Sequential chemoradiation therapy
TSH: Thyroid-stimulating hormone
