# Supplementary material for: Subsequent thyroid disorders associated with treatment strategy in head and neck cancer patients: a nationwide cohort study
Source: BMC Cancer. 2019 May 16;19:461. doi: 10.1186/s12885-019-5697-y (PMC6524259; doi:10.1186/s12885-019-5697-y)
Supplement: Supplementary file 1 — Figure S1. The cumulative incidence of subsequent thyroid disorders in PTE and PTE+ ND groups were present as solid and dotted curve, respectively. In our study design, the subjects with thyroid disorder occurred within one year after allocation were excluded, which is to prevent ill-defined cause-effect relationship, leading to no outcome obtained in the first year. (DOCX 219 kb) [file 12885_2019_5697_MOESM1_ESM.docx]

Figure S1. Cumulative incidence of thyroid disorders in patients who underwent PTE or PTE+ND.


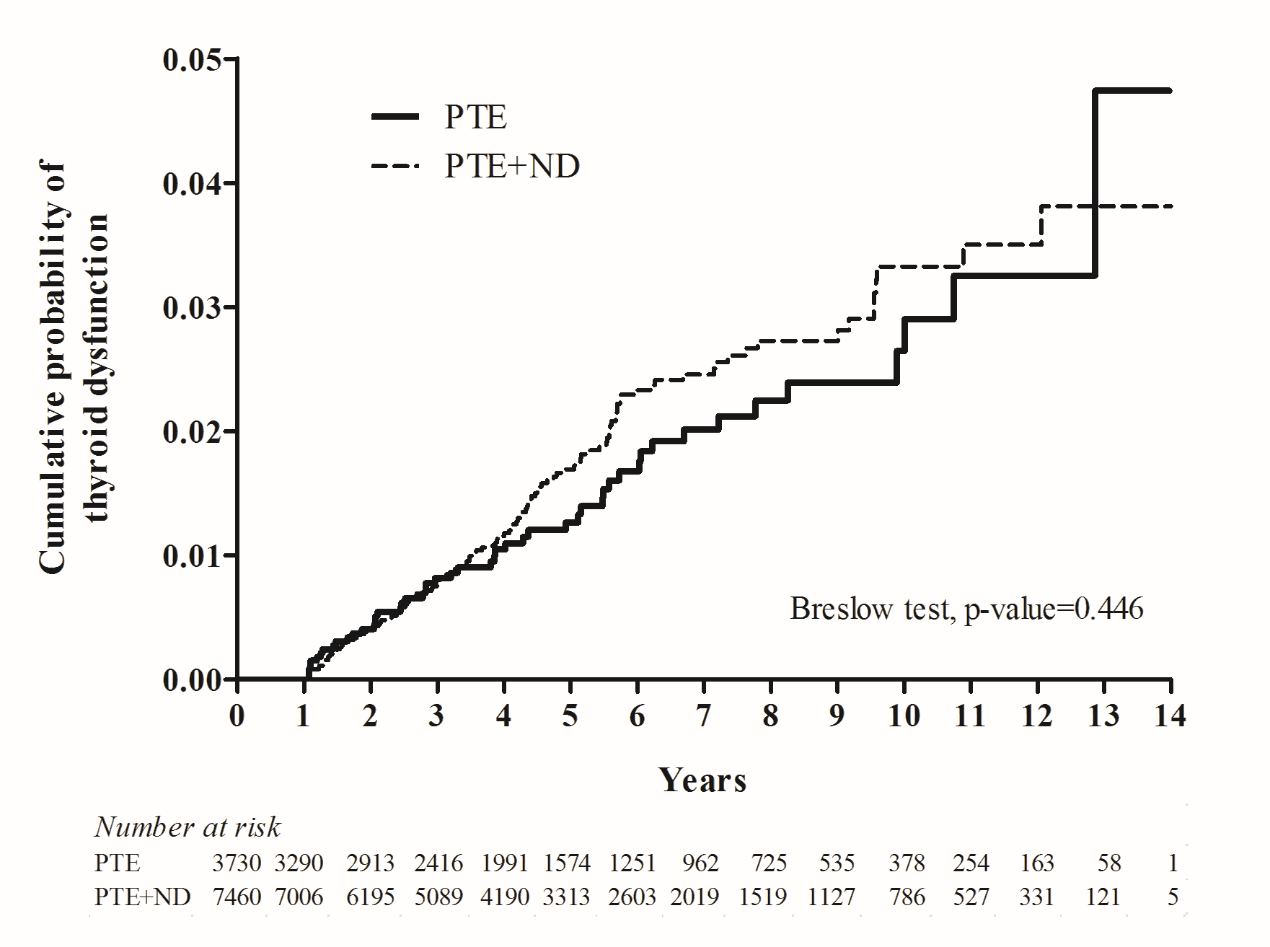


PTE, primary tumor excision; ND, neck dissection.
