# Supplementary material for: Subsequent thyroid disorders associated with treatment strategy in head and neck cancer patients: a nationwide cohort study
Source: BMC Cancer. 2019 May 16;19:461. doi: 10.1186/s12885-019-5697-y (PMC6524259; doi:10.1186/s12885-019-5697-y)
Supplement: Supplementary file 2 — Tables S1 and S2. The Table S1 showed the similar observation time in RT and CCRT groups of subjects received PTE + ND initially. In Table S2, subjects with higher age level distributed in RT group than in CCRT group. Both tables explained the RT group had higher risk of subsequent thyroid disorder than CCRT in subjects who received PTE + ND initially didn’t cause by longer follow-up time but by older age level distribution. (DOCX 16 kb) [file 12885_2019_5697_MOESM2_ESM.docx]

**Table S1** The mean observation time for patients who initially underwent PTE+ND in the RT only and CCRT groups.

|  | RT Only  (n = 597) | |  | CCRT  (n = 1110) | |
| --- | --- | --- | --- | --- | --- |
|  | Mean | SD |  | Mean | SD |
| Observation time (days) | 1706.70 | 1295.20 |  | 1694.56 | 1160.10 |

PTE, primary tumor excision; ND, neck dissection; RT, radiotherapy; CCRT, concomitant chemoradiation therapy; SD, standard deviation.

**Table S2** The number and proportion of patients who initially underwent PTE+ND in the RT only and CCRT groups, stratified by age.

|  | RT Only  (n = 597) | | CCRT  (n = 1110) | |  |
| --- | --- | --- | --- | --- | --- |
|  | N | % | N | % | p-value |
| Age (Mean ± SD) | 58.82 ± 12.71 | | 52.39 ± 10.84 | | < 0.001 |
| 20‒44 | 82 | 13.74 | 252 | 22.70 | < 0.001 |
| 45‒59 | 205 | 34.34 | 578 | 52.07 |  |
| ≥ 60 | 310 | 51.93 | 280 | 25.23 |  |

PTE, primary tumor excision; ND, neck dissection; RT, radiotherapy; CCRT, concomitant chemoradiation therapy; SD, standard deviation.
